# Lavender essential oil induces oxidative stress which modifies the bacterial membrane permeability of carbapenemase producing *Klebsiella pneumoniae*

**DOI:** 10.1038/s41598-019-55601-0

**Published:** 2020-01-21

**Authors:** Shun-Kai Yang, Khatijah Yusoff, Warren Thomas, Riaz Akseer, Maryam Sultan Alhosani, Aisha Abushelaibi, Swee-Hua-Erin Lim, Kok-Song Lai

**Affiliations:** 1 Health Sciences Division, Abu Dhabi Women’s College, Higher Colleges of Technology, 41012 Abu Dhabi, United Arab Emirates; 2 Department of Microbiology, Faculty of Biotechnology and Biomolecular Sciences, Universiti Putra Malaysia, 43400 Serdang, Selangor Malaysia; 3 Perdana University-Royal College of Surgeons in Ireland School of Medicine, Perdana University, MAEPS Building, 43400 Serdang, Selangor Malaysia

**Keywords:** Pharmacology, Antimicrobial resistance

## Abstract

Misuse of antibiotics in the clinical and agricultural sectors has caused the emergence of multidrug-resistant (MDR) *Klebsiella pneumoniae* which contributes a threat to human health. In this study, we assessed the feasibility of lavender essential oil (LVO) as an antimicrobial agent in combinatory therapy with meropenem in suppressing the growth of carbapenemase-producing *K*. *pneumoniae* (KPC-KP). Synergistic interactions between LVO and meropenem were detected, which significantly reduce the inhibitory concentration of both LVO and meropenem by 15 and 4-fold respectively. Comparative proteomic profiling identified a disruption in the bacterial membrane via oxidative stress that was indicated by loss of membrane and cytoplasmic proteins and the upregulation of oxidative regulators. As a proof of concept, zeta potential measurements showed a change in cell surface charge while outer membrane permeability measurement indicated an increase in membrane permeability following exposure to LVO. This was indicative of a disrupted outer membrane. Ethidium bromide influx/efflux assays demonstrated no significant efflux pump inhibition by LVO, and scanning electron microscopy revealed irregularities on the cell surface after exposure to LVO. Oxidative stress was also detected with increased level of ROS and lipid peroxidation in LVO-treated cells. In conclusion, our data suggest that LVO induced oxidative stress in *K*. *pneumoniae* which oxidizes the outer membrane, enabling the influx of generated ROS, LVO and meropenem into the bacterial cells, causing damage to the cells and eventually death.

## Introduction

*Klebsiella pneumoniae*, a Gram-negative rod-shaped bacterium is one of the leading causes of hospital acquired infections, and is especially implicated in bacterial pneumonia^1,2^. Misuse of antibiotics in the clinical and agricultural sectors has brought about the emergence of multidrug-resistant (MDR) *K*. *pneumoniae*, which is a threat to human health, especially in vulnerable groups such as neonates, the elderly and the immunocompromised^3,4^. Carbapenems are now considered as the last resort antibiotics for treating severe MDR *K*. *pneumoniae* infections. However, resistance towards carbapenems was soon documented in *K*. *pneumoniae* due to increased reliance upon this line of antibiotics^5,6^. Carbapenem-resistant *K*. *pneumoniae* produces carbapenemase, the most evolved β-lactamase currently in evidence, which can inactivate almost all classes of β-lactam antibiotics^7,8^. Other antibiotic resistance mechanisms such as the overproduction of class C beta-lactamases, expression of the MDR efflux pump or an ESBL coupled with bacterial membrane permeability defects are sufficient to confer a carbapenem resistance phenotype to *K*. *pneumonia*^9–11^. It has been reported that certain isolated carbapenem-resistant *K*. *pneumoniae* overexpress AcrAB pumps which remove a variety of antibiotics that penetrate the bacterial cell wall and membranes^12,13^. Another study confirmed reduction in expression of porin proteins which reduces membrane permeability, and so synergistically reduces the activity of antibiotics^13^. To further reduce the vulnerability of the bacterial membrane, carbapenem-resistant *K*. *pneumoniae* produce a modified capsule and lipopolysaccharide (LPS), which make up the outer surface of Gram-negative bacteria. Leung *et al*. (2017) showed that *K*. *pneumoniae* alters the composition of lipid A in LPS chains via hydroxylation, glycosylation and palmitoylation, conferring resistance to antimicrobial peptide, colistin^14^. To date, *K*. *pneumoniae* has evolved to become a formidable pathogen and serves as a major challenge in the clinical setting.

In order to mitigate problems caused by antibiotic resistance, much effort had been directed toward the development of “mining” strategies for the discovery of novel antimicrobial agents. This discovery approach has included the investigation of natural products due to their great diversity and relative abundance. Studies have shown that natural products such as plant essential oils (cinnamon bark, peppermint, tea tree, etc.) have tremendous potential as an antimicrobial resource due to their effective bactericidal action against a variety of bacterial pathogens^15–17^. To help control the emergence of novel antimicrobial resistance, alternative therapeutic regimes including combinatory therapy is often recommended and preferred, whereby two or more antimicrobial agents combining different modes of action are prescribed to a patient. Such a strategy reduces the possibility of enhanced resistance developing as suggested by Bassetti and Righi^18^. Combinatorial therapy can revive the efficacy of previous generations of antibiotics in the treatment of severe bacterial infections; this significantly increases the available treatment options^19^. Evidence from multiple studies has suggested that essential oil disrupts the bacterial membrane, eventually killing the target bacteria. For example, black pepper, cinnamon bark, clove basil and oregano essential oils were all reported to disrupt the bacterial membrane^20–23^. Despite the great potential of essential oils in mitigating antibiotic resistance, there has not been a study elucidating the mechanism behind the membrane disruption yet.

Lavender essential oil (LVO) is a popular essential oil commonly used in aromatherapy and also as an additive in various complementary medicine and cosmetic products. Throughout history, products of *Lavandula* spp have been used as therapeutic agents due to their antibacterial, anti-depressive, anti-inflammatory, carminative and sedative properties^24^. The antimicrobial activity of LVO against bacteria and fungi has long been established. However, few studies have been carried out to elucidate the mechanism of LVO action in order to capitalize on its application in clinical settings. Our current study was performed to assess the combinatory effects of LVO with meropenem, and also to elucidate the mechanism by which LVO acts against carbapenemase-producing *K*. *pneumoniae* (KPC-KP). The bactericidal activity and combinatory effects of LVO and meropenem were first determined followed by killing kinetics. The mode of action of LVO on KPC-KP was determined via comparative proteomic using nano liquid chromatography tandem mass spectrometry (LC-MS/MS) to elucidate the overall effects of LVO exposure on the proteome of KPC-KP cells. Quantitative and qualitative membrane integrity assays, specifically the zeta potential measurement, outer membrane permeability assay, ethidium bromide accumulation assay and scanning electron microscopy were performed. Ultimately, this study was undertaken to explain the antimicrobial mechanism of LVO that would pave the way for future potential drug discovery from LVO.

## Results

### Antimicrobial and combinatory activity LVO and meropenem

**Figure 1:**
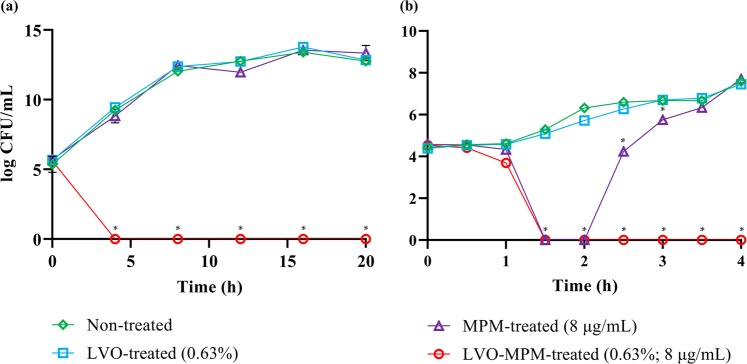
Time-kill study of KPC-KP cells challenged with LVO (0.63%) and meropenem (8 µg/mL), alone and in combination at 4 hour interval for 20 hours (**a**) and at 30 minutes interval for 4 hours (**b**). Data represents mean and standard error of mean of three independent experiments. Data in (**a** and **b**) were analyzed by one-way ANOVA at each time point; *p < 0.05.

The minimum inhibitory concentration (MIC) and combinatory activity of LVO with meropenem were determined using the resazurin microplate and checkerboard assays. The MIC of LVO and meropenem are 10% (v/v) and 32 μg/mL respectively (Table 1). However, when combined, both the MIC of LVO and meropenem was reduced significantly, from 10% (v/v) to 0.63% (v/v) for LVO and from 32 μg/mL to 8 μg/mL for meropenem. This combination yielded a synergistic reaction with combined fractional inhibitory concentration index (FICIc) value of 0.3125. The killing kinetics of LVO and meropenem were evaluated, alone and in combination at their sub-inhibitory concentration (LVO, 0.63%; meropenem, 8 μg/mL) by performing time kill analysis. A complete killing profile of KPC-KP cells treated with a combination of LVO and meropenem was observed after 4 h when viable count analysis was performed at 4 h intervals over 20 h (Fig. 1a). When time kill analysis was repeated at 30 min intervals over 4 h to obtain a more precise killing time, it was observed that only 1.5 h was required to obtain a complete killing profile for KPC-KP cells treated with combination of LVO and meropenem (Fig. 1b). The sub-inhibitory concentration of LVO (0.63%) and meropenem (8 μg/mL) administered alone did not affect KPC-KP cells viability.

**Table 1:** MIC and fractional inhibitory concentration (FIC) of LVO and meropenem against KPC-KP.

Components	KPC-KP				Type of interaction
	MIC	FIC	FICI	FICI_c_	
LVO (%)	10	0.63	0.06	0.31	Synergistic
Meropenem (µg/mL)	32	8	0.25		

FICI refers to the fractional inhibitory index. FICI_c_ ≤ 0.5, synergistic; FICI_c_ > 0.5–4.0, additive; FICI_c_ > 4.0, antagonistic.

### Comparative proteome profiling of KPC-KP cells

**Figure 2:**
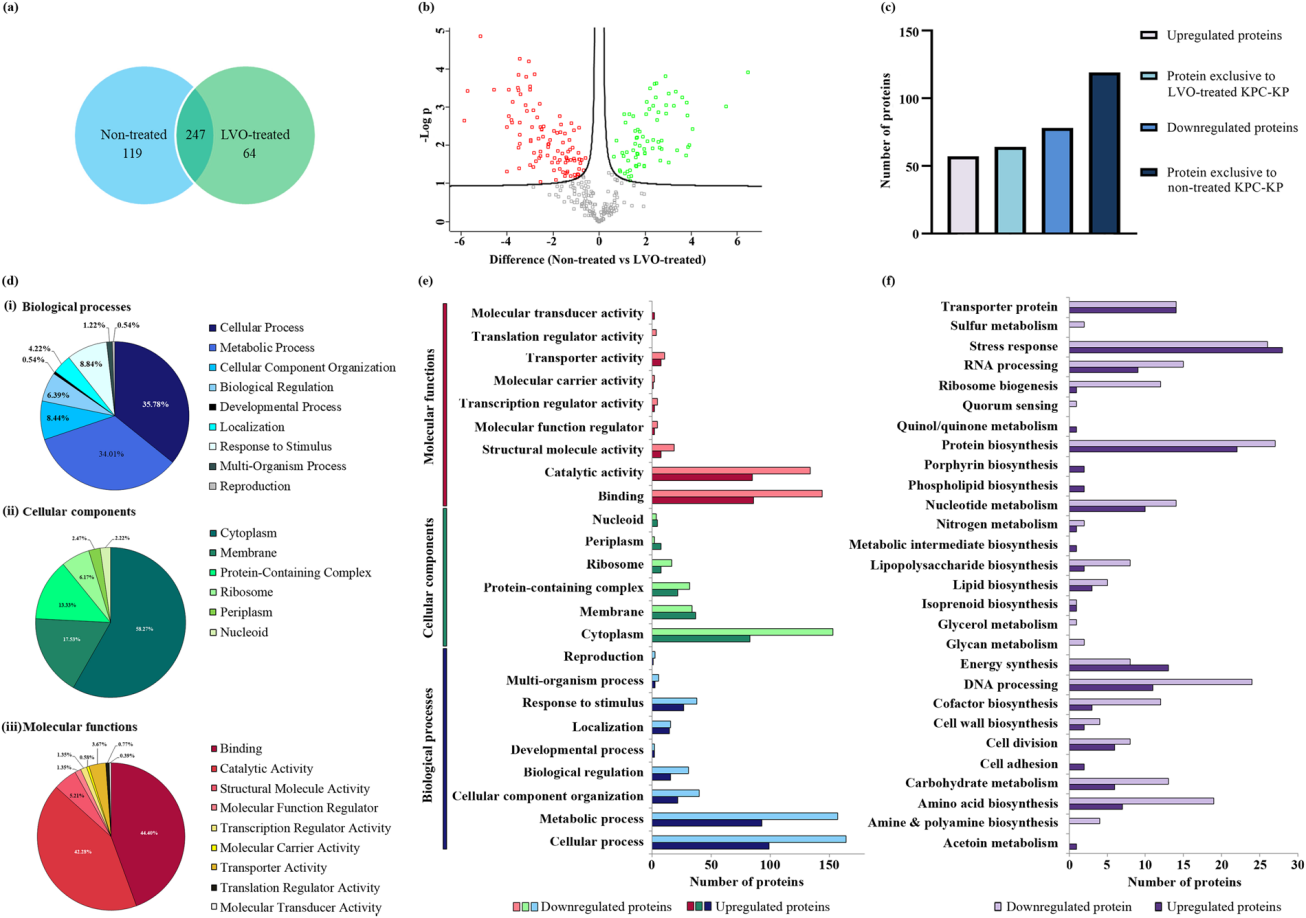
Comparative proteomic analysis between non-treated and LVO-treated KPC-KP cells. (**a**) Venn diagram of the total protein obtained from non-treated and LVO-treated KPC-KP cells. (**b**) Volcano plot showing up- (designated green square) and downregulated (designated red square) proteins of the LVO-treated KPC-KP cells. (**c**) The total numbers of exclusive, up-regulated and down-regulated proteins. (**d**) Gene ontology (GO) analysis in terms of biological processes (i), cellular components (ii) and molecular functions (iii) of identified proteins and their relative abundance (**e**), of LVO-treated KPC-KP cells. (**f**) KEGG pathway analysis of differentially expressed proteins in LVO-treated KPC-KP cells.

Comparative proteomic analysis was carried out between non-treated and LVO-treated KPC-KP cells in three independent experiments using Perseus Software v1.6.0.7 (Max Planck Institute of Biochemistry). Pearson correlation values between non-treated and LVO-treated KPC-KP were of high confidence, indicating the two treatment group are linearly related (Supplementary Fig. S1). In addition, principle component analysis revealed good separation between the two treatment groups, indicating significant changes in the proteomic abundance between each group (Supplementary Fig. S2). A total of 366 proteins from the non-treated KPC-KP cells and 311 proteins from the LVO-treated KPC-KP cells were successfully identified (Fig. 2a). Both groups shared a total of 247 similar proteins. From the analysis, 57 proteins were upregulated and 78 proteins were downregulated following exposure to LVO (Fig. 2b,c). Furthermore, 64 proteins were exclusive to the LVO-treated cells and 119 proteins were exclusive to the non-treated cells (Fig. 2c). Under LVO exposure, the proteins with the greatest increase in abundance were NAD(P)H dehydrogenase (quinone) (5.2-fold), β-lactamase SHV-11 (4.6-fold) and ribosome-recycling factor (4.5-fold) whereas most reduced abundance was detected for NADH-quinone oxidoreductase subunit B (−5.6-fold), outer membrane protein A (−5.2-fold) and mannitol-1-phosphate 5-dehydrogenase (−4.1-fold) (Table 2). The full record of proteomic abundance changes is as detailed in Supplementary Spreadsheet S1. Four upregulated proteins namely phosphopentomutase (deoB), 3-hydroxydecanoyl-[acyl-carrier-protein] dehydratase (fabA), uridine phosphorylase (udp) and cell division protein ZapB (zapB) were selected for proteome validation using qRT-PCR and results showed a similar trend with the proteomic profile (Supplementary Fig. S3). The list of primers used is also attached in Supplementary Table S1.

**Table 2:** Top 20 up- and down-regulated proteins of significant difference in KPC-KP cells exposed to LVO.

No	Proteins	Gene name	Uniprot accession no.	General function	Cellular component	Fold change
**Upregulated proteins**						
1	NAD(P)H dehydrogenase (quinone)	*wrbA*	B5XXP0	Stress response	Membrane	5.15458
2	Beta-lactamase SHV-11	*blaSHV-11*	P37323	Stress response	Cytoplasm	4.62696
3	Ribosome-recycling factor	*frr*	B5Y1J9	Protein biosynthesis	Cytoplasm	4.54865
4	Phosphopentomutase	*deoB*	B5Y275	Metabolic intermediate biosynthesis	Cytoplasm	4.05602
5	Diacetyl reductase [(S)-acetoin forming]	*budC*	Q48436	Acetoin metabolism	Cytoplasm	4.01896
6	Cell division protein ZapB	*zapB*	A6TFR0	Cell division	Membrane	3.66352
7	Uridine phosphorylase	*udp*	P52671	Nucleotide metabolism	Cytoplasm	3.41065
8	2,3,4,5-tetrahydropyridine-2,6-dicarboxylate N-succinyltransferase	*dapD*	B5Y1K5	Cell wall biosyntheiss	Cytoplasm	3.27497
9	Elongation factor Ts	*tsf*	A6T4X2	Protein biosynthesis	Cytoplasm	3.19873
10	Methionine–tRNA ligase	*metG*	A6TBK7	Protein biosynthesis	Cytoplasm	2.92754
11	Carbapenem-hydrolyzing beta-lactamase KPC	*bla*	Q9F663	Stress response	Cytoplasm	2.90361
12	Adenylate kinase	*adk*	A6T5N7	Nucleotide metabolism	Cytoplasm	2.87125
13	DNA protection during starvation protein	*dps*	A6T6Q6	DNA processing	Cytoplasm	2.87
14	Glucose-6-phosphate isomerase	*pgi*	A6TGT4	Energy synthesis	Cytoplasm	2.78288
15	Xanthine phosphoribosyltransferase	*gpt*	A6T532	Nucleotide metabolism	Membrane	2.72207
16	Autonomous glycyl radical cofactor	*grcA*	A6TCJ1	Stress response	Cytoplasm	2.71508
17	Purine nucleoside phosphorylase DeoD-type	*deoD*	B5Y274	Nucleotide metabolism	Membrane	2.54311
18	2,3-bisphosphoglycerate-dependent phosphoglycerate mutase	*gpmA*	B5XZB2	Energy synthesis	Cytoplasm	2.43127
19	50S ribosomal protein L7/L12	*rplL*	A6TGN9	Protein biosynthesis	Ribosomal protein	2.32274
20	3-hydroxydecanoyl-[acyl-carrier-protein] dehydratase	*fabA*	A6T748	Fatty acid biosynthesis	Cytoplasm	2.26672
**Downregulated proteins**						
21	NADH-quinone oxidoreductase subunit B	*nuoB*	A6TBX3	Transporter protein	Membrane	−5.59153
22	Outer membrane protein A	*ompA*	P24017	Transporter protein	Membrane	−5.24924
23	Mannitol-1-phosphate 5-dehydrogenase	*mtlD*	A6TFJ4	Manitol metabolism	Cytoplasm	−4.12857
24	ATP synthase subunit c	*atpE*	B5XZL9	Energy synthesis	Membrane	−3.95817
25	60 kDa chaperonin	*groL*	A6TH53	Stress response	Cytoplasm	−3.92018
26	ATP synthase subunit b	*atpF*	B5XZM0	Energy synthesis	Membrane	−3.89086
27	NADH-quinone oxidoreductase subunit A	*nuoA*	A6TBX4	Transporter protein	Membrane	−3.74012
28	D-erythrose-4-phosphate dehydrogenase	*epd*	A6TDT2	Cofactor biosynthesis	Cytoplasm	−3.51524
29	Exodeoxyribonuclease 7 small subunit	*xseB*	B5Y0W9	DNA processing	Cytoplasm	−3.47431
30	ATP phosphoribosyltransferase	*hisG*	B5XPE8	Protein biosynthesis	Cytoplasm	−3.3997
31	ATP synthase gamma chain	*atpG*	A6TG37	Energy synthesis	Membrane	−3.39661
32	ATP synthase subunit beta	*atpD*	A6TG36	Energy synthesis	Membrane	−3.32617
33	DNA-directed RNA polymerase subunit beta	*rpoB*	A6TGP0	DNA processing	Cytoplasm	−3.20933
34	DNA-directed RNA polymerase subunit alpha	*rpoA*	A6TEU8	DNA processing	Cytoplasm	−3.19363
35	Chaperone protein DnaJ	*dnaJ*	A6T4F5	Stress response	Cytoplasm	−3.15398
36	50S ribosomal protein L35	*rpmI*	B5XQC8	Protein biosynthesis	Ribosomal protein	−3.12775
37	Leucine-responsive regulatory protein	*lrp*	P37424	DNA processing	Cytoplasm	−3.05971
38	Outer membrane protein C	*ompC*	Q48473	Transporter protein	Membrane	−3.03548
39	ATP synthase epsilon chain	*atpC*	A6TG35	Energy synthesis	Membrane	−3.03454
40	Lipoyl synthase	*lipA*	B5XZS6	Protein biosynthesis	Cytoplasm	−3.02594

All identified proteins were subjected to gene ontology (GO) analysis, classified into three categories namely, biological processes, cellular components and molecular functions. Changes in protein abundance for each group are shown in Fig. 2d,e, followed by KEGG pathway analysis to determine the overall effect of LVO on the proteome of the KPC-KP cells (Fig. 2f). In terms of biological processes, the majority of the proteins identified were categorized under cellular and metabolic processes (35.78% and 34.01%) followed by cellular component organization and response to stimulus (8.44% and 8.84%) as shown in Fig. 2d-i. With regards to cellular components, the majority of the proteins identified were categorized under cytoplasm and membrane (58.27% and 17.53%) followed by protein-containing complexes and ribosomal proteins (13.33% and 6.17%; Fig. 2d-ii). Molecular function categorizations of the identified proteins showed that the majority of the proteins were involved in binding and catalytic activity (44.4% and 42.28%; Fig. 2d-iii). According to the KEGG pathway analysis (Fig. 2f), proteins related to stress responses were most affected by exposure to LVO, followed by protein biosynthesis and DNA processing proteins. A detailed categorization of the proteomic profile can be found in Supplementary Spreadsheet S1.

### Disruption of bacterial membrane via oxidative stress

**Figure 3:**
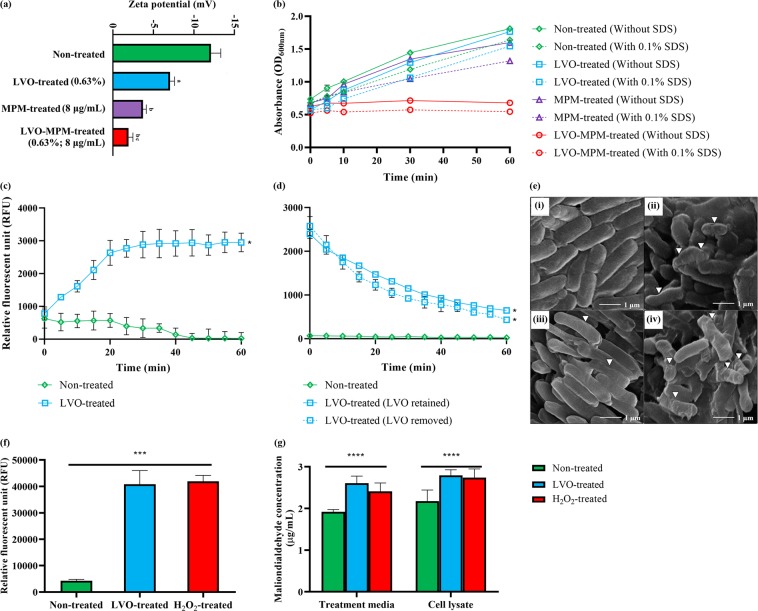
LVO disrupts bacterial membrane of KPC-KP cells by generating oxidative stress. (**a**) Membrane potential of KPC-KP cells treated with LVO (0.63%), meropenem (8 μg/mL) and combination of both. (**b**) Outer membrane permeability of KPC-KP cells exposed to 0.1% SDS or saline after treatment with LVO (0.63%), meropenem (8 μg/mL) and combination of both. (**c**) Ethidium bromide accumulation assay with non-treated and LVO-treated cells exposed to 1 µg/mL ethidium bromide for 60 minutes at 5 minute intervals followed by (**d**) ethidium bromide efflux assay with the removal of ethidium bromide and fluorescent reading taken for 60 minutes at 5 minute intervals. (e) Scanning electron micrograph of non-treated KPC-KP cells (i), cells treated with LVO (0.63%; ii), meropenem (8 μg/mL; iii) and combination of both (iv). Oxidative stress assessment by ROS measurement using DCF-DA (**f**) and lipid peroxidation assay (**g**). Data were analyzed by one-way ANOVA; *^,a,b and c^p < 0.05, ***p < 0.001, ****p < 0.0001.

As shown in Fig. 2e, a total of 153 proteins found within the bacterial cytoplasm and 34 bacterial membrane proteins in KPC-KP cells exposed to LVO had reduced abundance when compared to the non-treated cells (Supplementary Spreadsheet S1). This suggests that a disrupted bacterial membrane led to the loss of cytoplasmic proteins. To confirm this observation, we performed membrane-related quantitative and qualitative assays including measurement of membrane potential, outer membrane permeability assay, influx/efflux assay and scanning electron microscopy (Fig. 3). The membrane potential measurement determines the bacterial surface charge by detecting the mobility of bacterial cells in the presence of an electrophoretic force, under condition where the pH and salt concentration are standardized. The zeta potential of the non-treated KPC-KP cells was −12.1 mV while treated cells had significantly more positive values (Fig. 3a). KPC-KP cells treated separately with either LVO or meropenem alone had zeta potential values of −7.05 mV and −3.71 mV, respectively, while the combination of both agents resulted in a more positive zeta potential value of −1.91 mV (Fig. 3a).

The outer membrane permeability assay was also performed to investigate the bacterial membrane permeabilization action of LVO. Figure 3b compares the growth of the KPC-KP cells; control (only MHB broth supplemented with 10% Tween 80), treated with LVO (0.63%) and meropenem (8 μg/mL) alone and in combination, through differential absorbance at 600 nm pre- and post-exposure to 0.1% sodium dodecyl sulfate (SDS) solution. The control group, LVO-treated and meropenem-treated cells showed normal growth in the absence of SDS with the control group having a slightly higher growth rate than cells treated with LVO and meropenem. In the presence of SDS, there was a reduction in the growth rate, as even a low concentration of SDS (0.1%) exerts stress to the growing cells. However, bacterial cells treated with the combination of LVO and meropenem, in the absence or presence of 0.1% SDS, had a lower absorbance compared to those of controls and treated with LVO and meropenem. In comparison to the non-treated cells, a significant drop in the absorbance can be detected when LVO-treated cells were exposed to 0.1% SDS, indicating the ability of LVO to enhance the SDS-induced cell lysis, and slowing cell growth. A combination of LVO with meropenem had the lowest absorbance reading, with a decreasing trend, among four groups, especially when exposed to 0.1% SDS.

An influx/efflux assay was performed using ethidium bromide as a probe to determine membrane permeability and the functionality of the bacterial efflux system. As shown in Fig. 3c, KPC-KP cells exposed to ethidium bromide (1 µg/mL) alone had decreased fluorescent intensity overtime while cells exposed to both ethidium bromide (1 µg/mL) and LVO (0.63%) showed a significant and gradual increase in the fluorescent intensity. This indicated that there was an influx of ethidium bromide in the presence of LVO. An efflux assay was performed immediately after the influx assay ended by removing ethidium bromide and LVO from each treatment group (Fig. 3d). KPC-KP cells treated with ethidium bromide alone during the influx assay had similar decrease in the fluorescent unit overtime after the removal of ethidium bromide, indicating an active efflux system which continuously removed ethidium bromide. Cells treated with ethidium bromide and LVO were divided into two parts, one with ethidium bromide removed and the other with both ethidium bromide and LVO removed. Removal of ethidium bromide and/or LVO from KPC-KP cells caused the cells to exhibit active efflux, as indicated by the rapid decrease of fluorescent intensity (Fig. 3d).

Scanning electron microscopy was performed to determine the effect of LVO on the overall morphology of KPC-KP cells. Non-treated KPC-KP cells were typical rod-shaped bacteria with smooth surfaces (Fig. 3e(i)). Upon exposure to LVO at 0.63%, irregularities in the surface of the bacteria can be observed, with the presence of a corrugated cell envelope (Fig. 3e(ii)). KPC-KP cells treated with meropenem (8 μg/mL) alone showed minor irregularities on the surface membrane of the bacteria (Fig. 3e(iii)). Combinatory treatment with both LVO (0.63%) and meropenem (8 μg/mL) produced a more extensive distortion and corrugation in the cell envelope of the bacteria as indicated by the arrows in Fig. 3e(iv).

Presence of oxidative stress implied from comparative proteomic profiling analysis (Supplementart spreadsheet 1), suggests the presence of reactive oxygen (ROS), which leads to lipid peroxidation and eventually membrane disruption. To validate these phenomena, lipid peroxidation assay and measurement of ROS were performed. The level of ROS quantified in LVO-treated KPC-KP cells was significantly higher than the non-treated cells as shown in Fig. 3f, indicating high level of ROS generated in KPC-KP cells upon exposure to LVO. From Fig. 3g, the concentration of malondialdehyde (MDA) quantified in LVO-treated KPC-KP cells are also significantly higher than those in the non-treated cells, indicating the presence of lipid peroxidation of cells upon exposure to LVO, explaining the observed disruption of membrane in KPC-KP cells.

### Determination of the chemical composition of LVO using gas chromatography mass spectrometry (GC-MS)

A total of 31 chemical compounds were identified from LVO using GC-MS analysis (Supplementary Table S2). Linalyl anthranilate and linalool were the major compounds, with an overall percentages of 45.9% and 34.5% respectively, followed by β-caryophyllene and borneol at 2.4% and 1.9% respectively. Additionally, we also found that 10 out of 31 compounds were reported as antioxidants while 4 were pro-oxidants (Supplementary Table S2).

## Discussion

The development of antibiotic resistance by human infectious pathogens has become a major clinical challenge, impeding the progress of patient recovery and even increasing mortality rates over the course of infection. The problem is further complicated by the emergence of carbapenem-resistant bacterial infection in the last decade. This resistance phenomenon is particularly concerning as carbapenem is a last resort group of beta lactamase antibiotics which possesses the broadest spectrum of activity and highest potency amongst all β-lactam antibiotics^25^. Thus, discovery of novel antimicrobials is going to be crucial, and has spurred many research groups to look into natural products with the hopes of overcoming the resistance problem. Essential oils consist of a mixture of many compounds which would allow identification of novel antimicrobial agent for clinical application in the near future. The bioactivity of essential oils has been documented in many studies; however, little is known about the mechanism by which essential oils act against bacteria. Thus, this study was carried out to investigate the mode of action of essential oil in bacterial killing by using LVO, and KPC-KP as a model organism. We first determined the bioactivity of LVO and meropenem against KPC-KP cells and found that KPC-KP cells were relatively resistant towards the two agents with high MIC values. A combination of LVO and meropenem, however, successfully reduced the individual MIC values significantly to 0.63% for LVO and 8 μg/mL for meropenem, indicating synergism in action. Similarly, a study done by Yap *et al*. (2013) also suggested essential oils, including LVO reacted synergistically with several antibiotics when used against a panel of bacteria^16^.

To further elucidate the overall effect of LVO on KPC-KP cells, we took a proteomic approach by comparing the proteome of both non-treated and LVO-treated KPC-KP cells via LC-MS/MS. The comparative proteome profile showed disruption in the bacterial membrane with 153 cytoplasmic proteins and 34 membrane proteins reduced in abundance or lost following the exposure to LVO. This indicates an intracellular leakage which might be attributed to membrane disruption as previously proposed in other studies^20,26^. As proof of concept, we performed various membrane related assays, quantitatively and qualitatively. Zeta potential measurement showed that LVO had affected the bacterial membrane significantly by increasing the overall surface charges of KPC-KP cells, indicating a permeable membrane. The negative surface charge of KPC-KP cell is linked to LPS abundance which forms the outer membrane of the bacteria. An increase in the overall surface charges indicates loss of LPS.

To determine the permeability of the bacterial membrane, both SDS (0.1%) and ethidium bromide (1 µg/mL) were used as a probe; these would only enter the intracellular region of bacterial cells when the membrane is damaged^21,27,28^. From the outer membrane permeability assay, KPC-KP cells exposed to LVO had higher permeability for SDS (0.1%) when compared to the non-treated cells, significantly impeding the growth of the cells and eventually killing them. This again indicated a disrupted bacterial membrane which facilitated the disruptive action of SDS and killing of the cells. The efflux system inhibition can occur through several mechanisms: by antagonizing the energy synthesis which powers the efflux activity; by acting as a competitive or non-competitive substrate which binds to the efflux pump or by causing a conformational change to the efflux pump^29^. The ethidium bromide assay allows the determination of whether LVO affects the activity of the efflux system by competitive or non-competitive substrate binding or by changing the conformation of the pump subunits. Interestingly, ethidium bromide influx and efflux assays showed that influx of ethidium bromide into the KPC-KP cells after treatment with LVO. Subsequently, KPC-KP cells demonstrated the ability to actively remove ethidium bromide in the presence of LVO. This indicated that LVO affects mainly the membrane permeability of KPC-KP cells without affecting the efflux system. A previous study by Limaverde *et al*. (2017) showed that *Chenopodium ambrosioides* essential oil act as an effective efflux pump inhibitor^30^. The compound responsible for the inhibition was α-terpinene^30^. A consolidating review study of 28 plant-derived compounds identified compounds such as α-pinene, catechol, eugenol acetate and linoleic acid as efflux pump inhibitors^31^. Our GC-MS analysis of LVO (Table S3) did not identify any compounds that had been described as plant-derived efflux pump inhibitors, which explains the absence of efflux inhibition activity in the ethidium bromide efflux assay.

Scanning electron microscopy showed that exposure to LVO had significant effects on the bacterial surface, corrugating the bacterial outer membrane of KPC-KP cells. Cells exposed to LVO only and cells treated with a combination of LVO and meropenem shared similar corrugation of the cell surface (Fig. 3e). However, time-kill analysis (Fig. 1) found that only the combination killed the KPC-KP cells. LVO alone at sub-inhibitory concentrations only caused slight disruption to the outer membrane of KPC-KP cells (Fig. 3e). A disrupted outer membrane is insufficient to kill KPC-KP cells as the cell walls still remain intact, preventing bacterial lysis via osmotic pressure. However, LVO in combination with meropenem killed the cells as meropenem disrupts cell wall biosynthesis. The simultaneous disruption of both the outer membrane and cell wall may kill the bacterial cells through the leakage of intracellular contents and osmotic pressure. This further demonstrates the use of essential oil in combinatory therapy as a promising alternative in mitigating antimicrobial resistance in the clinical settings, in a sense that the disrupted bacterial membrane facilitates the uptake of antibiotics into the bacterial cell, so enhancing the efficacy of antibiotics. Studies by Rao *et al*. (2017) and Yang *et al*. (2017) have similarly revealed that the combinatory action of *Geophila repens* and cinnamon bark essential oil greatly reduces the MIC of antibiotics used against pathogenic strains of *Pseudomonas aeruginosa* and *K*. *pneumoniae*^15,32^.

Essential oils disrupt bacterial membranes in this and in previous studies^15,33^. The exact mechanism of how bacterial membrane disruption occurs during exposure to essential oil has yet to be elucidated. In our proteomic KEGG pathway analysis, 28 proteins related to the stress response had increased abundance following the exposure to LVO. Of the 28 upregulated proteins, 15 were oxidative stress-related proteins which are involved in the repair of genetic material and proteins (Supplementary Spreadsheet S1). Additionally, we also found that the majority of proteins involved in ribosome biogenesis, and DNA and RNA processing had reduced abundance when compared to the non-treated KPC-KP cells. It is known that ribosomal and genetic material processing proteins are relatively sensitive to oxidative stress^34,35^. For instance, oxidative damage can induce base substitution, addition, deletion and other mutation in nucleic acids which leads to the formation of non-functioning proteins^36^. In addition, oxidative damage also affects proteins, especially ribosomal proteins due to the affinity of ROS for RNA^37^. Studies showed that oxidation of RNA indirectly causes damage to ribosomal RNA, via covalent modification, leading to defective protein synthesis^34,37^. The evidence obtained from the proteomic analysis reveals that LVO induces oxidative stress in KPC-KP cells and is further proven by the measurement of ROS using 2′, 7′–dichlorofluorescin diacetate (DCF-DA) in this study (Fig. 3f). ROS generation can be quantified via the reaction between DCF-DA, esterase enzyme and ROS species^38^. LVO-treated cells had significantly higher amount of ROS when compared to non-treated cells. Induction of oxidative stress is also known to affect the integrity of eukaryotic cell membranes. This is termed lipid peroxidation, but information on lipid peroxidation in the bacterial membrane is scarce^39,40^. Given the similarities between the membrane of prokaryotic and eukaryotic cells, we hypothesize that lipid peroxidation may occur similarly in both types of cells^41,42^. Lipid peroxidation is a self-propagating chain reaction which involves reactions between a ROS and membrane fatty acid, eventually destroying the membrane of a cell^43,44^. Interestingly, as revealed from the KEGG pathway analysis, we identified seven proteins involved in membrane biosynthesis, specifically lipid, LPS and phospholipid biosynthesis which showed increased abundance, inferring the activation of a repair mechanism in the disturbed bacterial membrane. In addition, lipid peroxidation assays performed in this study further supports our observation; LVO-treated KPC-KP cells had significantly higher amount of MDA when compared to non-treated cells (Fig. 3g). Lipid peroxidation assay measures the end product of lipid peroxidation, MDA, which reacts readily with thiobarbituric acid (TBA), forming a pink mixture, which can be quantified via spectrophotometer^38^. In contrast with our results, however, essential oils are known to have a large amount of antioxidant activity^45–47^. Our GC-MS data revealed that 10 out of 31 compounds in LVO were antioxidants (Table S3)^48–55^. The observed oxidative stress phenomena in this study can be explained by the presence of pro-oxidants in LVO. Pro-oxidants are defined as compounds which induce oxidative stress either by generating ROS or by inhibiting antioxidation systems^56^. Acetic acid, geranyl acetate, linalool and pyrrolidines were reported to be pro-oxidants which induce oxidative stress in numerous studies^54,57–59^. Mimica-Dukić *et al*. (2016) reported that a single compound can work both as an antioxidant and a prooxidant at the same time^60^. For example, acetic acid, linalool and pyrrolidines have all been reported to function both as antioxidants and pro-oxidants^48,53,57–59,61^. Such a scenario is feasible as the method used to determine the oxidative nature of compounds differ significantly in terms of reaction mechanisms, oxidant and target species^60^. Additionally, no studies have been performed on the majority of the compounds in LVO, suggesting that there may be more pro-oxidants yet to be discovered which may exist in essential oils.

**Figure 4:**
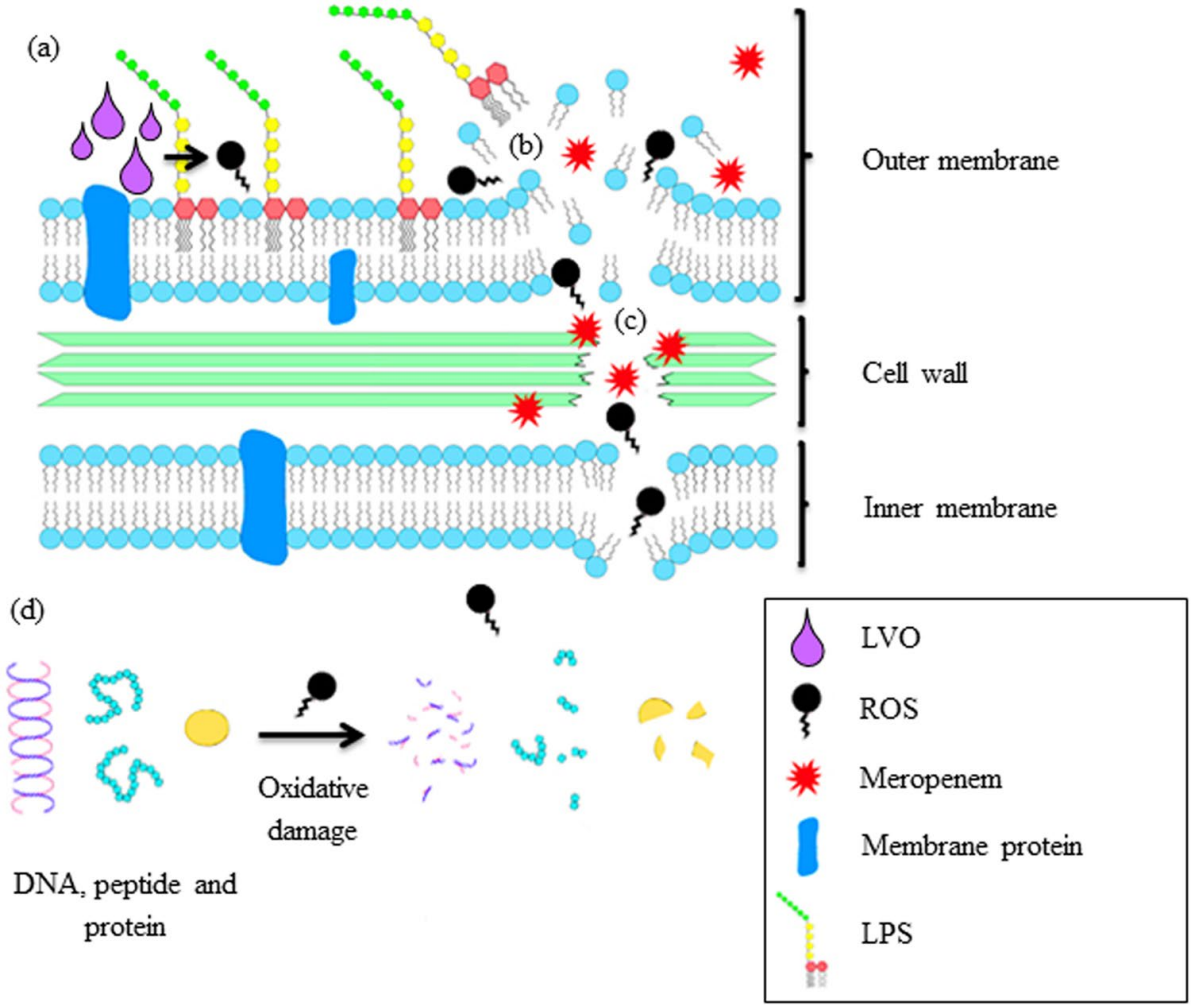
Proposed mode of action of LVO against KPC-KP cells. (**a**) LVO reacts with cell outer membrane forming ROS. (**b**) ROS oxidize outer membrane, causing a chain reaction which disrupts the outer membrane. (**c**) Influx of meropenem and ROS disrupts bacterial cell wall and inner membrane. (**d**) ROS reacts with genetic materials such as DNA and RNA and proteins, damaging them and eventually killing the cell.

Our study successfully showed LVO as promising antimicrobial extract which is suitable for novel antimicrobial agent identification which can be used in combinatory therapy, and may possibly be effective in reviving the efficacy of carbapenem and other antibiotics against other resistant bacteria. As illustrated in Fig. 4, we have also proposed the overall mode of action of LVO in disrupting the bacterial membrane via oxidative stress. Oxidative stress initiated by LVO when interacting with the bacterial membrane, creates a self-propagating reaction which progressively damages the membrane, making it more permeable. As revealed by the proteomic analysis, the loss of membrane and cytoplasmic proteins provided clues to the membrane disrupting ability of LVO that was further supported through membrane permeability assays and electron microscopy. We also showed that LVO had no effect on the inhibition of the efflux system that played a big part in antibiotic resistance in KPC-KP cells. The membrane disruptive ability of LVO may instead facilitate greater influx of meropenem and ROS into the cell, synergistically enhancing the killing of KPC-KP cells. In conclusion, LVO has great potential in reviving the efficacy of antibiotics in the clinical setting and our study has provided a platform for future studies to focus on the isolation of compounds (Table S3) responsible for the activity observed in LVO in this study.

## Methods

### LVO and meropenem

Lavender (*Lavandula angustifolia*) essential oil used in this study was supplied by Aroma Trading Ltd. (Milton Keynes, UK). Meropenem trihydrate was purchased from Sigma-Aldrich Corporation (St. Louis, MO, USA). A stock solution of 10 mg/mL stock meropenem solution was made by dissolving meropenem trihydrate in sterile distilled water.

### Bacterial strains and growth conditions

Carbapenem resistant KPC-producing *Klebsiella pneumoniae* BAA-1705 (KPC-KP) and *Escherichia coli* ATCC 25922 were purchased from American Type Culture Collection (ATCC, Manassas, VA, USA). Both bacterial strains were cultured and maintained on Mueller-Hinton agar (MHA; Sigma-Aldrich). Subsequently, a single colony was inoculated into Mueller-Hinton broth (MHB; Sigma-Aldrich) at 37 °C and shaking at 250 rpm for 16 h.

### MIC assay

The MIC assay was performed using broth microdilution as detailed in Yang *et al*.^15^. Tween 80, at final concentration of 10%, was incorporated into the MHB in order to enhance the solubility of LVO, and resazurin (7-hydroxy-3H-phenoxazin-3-one-10-oxide) at a final concentration of 0.02% was used to improve the visualization^15^. Two-fold dilutions were performed in each test well to yield final well volumes composed of 50 μL of test compound, 40 μL of bacterial suspension at approximately 1 × 10^5^ cfu/mL and 10 μL resazurin at a final concentration of 0.02%^15^. *E*. *coli* ATCC 25922 was used as an antibiotic (meropenem) positive control in this assay. Assays were performed in three independent replicate followed with incubation at 37 °C and shaking at 200 rpm for 20 h. The MICs of LVO and meropenem were determined qualitatively and quantitatively using the resazurin color change and relative fluorescence^15^.

### Checkerboard assay

The checkerboard assay was performed as detailed by Yang *et al*. (2017). Ten serial, two-fold dilutions of meropenem and five serial, two-fold dilutions of LVO were prepared to determine the combinatory effects of LVO and meropenem against KPC-KP cells^15^. Each well contained 25 μL of meropenem and 25 μL of LVO inoculated with 40 μL of bacterial suspension and 10 μL of resazurin to make final concentration of approximately 1 × 10^5^ cfu/mL and 0.02%^15^. The 96-well plates were then incubated at 37 °C with shaking at 200 rpm for 20 h. Combinatory relationship between LVO and meropenem was expressed in terms of fractional inhibitory concentration index (FICI) using the following formulas^15,16^:

$${\rm{FICI}}\,{\rm{of}}\,{\rm{LVO}}=\frac{{\rm{MIC}}\,{\rm{of}}\,{\rm{LVO}}\,{\rm{in}}\,{\rm{combination}}}{{\rm{MIC}}\,{\rm{of}}\,{\rm{LVO}}\,{\rm{alone}}}$$

$${\rm{FICI}}\,{\rm{of}}\,{\rm{meropenem}}=\frac{{\rm{MIC}}\,{\rm{of}}\,{\rm{meropenem}}\,{\rm{in}}\,{\rm{combination}}}{{\rm{MIC}}\,{\rm{of}}\,{\rm{meropenem}}\,{\rm{alone}}}$$

$${\rm{FICIc}}={\rm{FICI}}\,{\rm{of}}\,{\rm{LVO}}+{\rm{FICI}}\,{\rm{of}}\,{\rm{meropenem}}$$

$${\rm{FICIc}}\,\le 0.5,{\rm{synergistic}};{\rm{FICIc}}\, > 0.5-4.0,{\rm{additive}};{\rm{FICIc}}\, > 4.0,{\rm{antagonistic}}.$$

### Time kill assay

A standard inoculum of 1 × 10^5^ cfu/mL was used in the time kill analysis through viable colony forming unit counting^15^. The test concentrations of LVO and meropenem used were as determined from the previous checkerboard assay. The time kill analysis consisted of a non-treated control sample (inoculum with MHB supplemented with 10% Tween 80 at final concentration), samples treated with LVO (0.63%), samples treated with meropenem (8 μg/mL) and samples treated with the combination of LVO and meropenem (0.63% and 8 μg/mL)^15^. Each treatment had a final volume of 20 mL supplemented with 10% Tween 80 to enhance the solubility of LVO. Samples were incubated at 37 °C with shaking at 200 rpm. Immediately after inoculation, viable counting was performed every 4 h for 20 h. The measurement for viable counting was recorded half hourly if rapid killing was observed. A volume of 50 μL of samples were obtained and subjected to 100-fold dilution with 0.85% (w/v) saline and then plated onto Mueller-Hinton agar (MHA) and incubated at 37 °C for 16 h. The time kill analysis was performed in three independent replicate.

## Comparative Proteomic Analysis

### Whole cell protein extraction

KPC-KP cells were treated with LVO and meropenem with concentrations and treatment times determined from the checkerboard assay and time kill analysis. Non-treated and LVO-treated cultures had a final volume of 50 mL, supplemented with Tween 80 at final concentration of 10% to enhance the solubility of LVO. A standard inoculum of 1 × 10^5^ cfu/mL KPC-KP cells were used for treated and non-treated culture preparations. Cultures were incubated at 37 °C with shaking at 200 rpm for 16 h to obtain enough cells for protein extraction. Cell pellets from both treatment groups were obtained via centrifugation at 9000 rpm for 10 min; pellets were washed at least three times and resuspended in 500 μL cold protein extraction buffer (50 mM ammonium bicarbonate, 10 mM phenylmethylsulfonyl fluoride)^62^. Samples were then ultra-sonicated on ice at 20 amplitude for 10 cycles; each cycle consists of 10 sec of sonication followed by a 20 sec cooling period (Qsonica Sonicator Q55, Fischer Scientific, USA)^62^. Ultra-sonicated samples were centrifuged at 4 °C and 10,000 rpm for 1 h; supernatants were then collected and the protein concentration was quantified by Bradford assay. The protein concentration of each samples were standardized to 1 mg/mL for the subsequent proteomic analysis. Treatment was performed on three independent biological replicates to ensure the reproducibility of the experiment.

### Protein sample preparation

Approximately 100 μg of total protein was resuspended in 100 μL of 50 mM ammonium bicarbonate (pH 8.0)^62^. RapiGest surfactant (Waters Corporation, USA) was added to the extracted protein in equal parts at a final concentration of 0.05%^62^. The proteins from each sample were then concentrated to a volume of 100 µL using a Vivaspin™ column (GE Healthcare, USA) with a molecular weight cut-off (MWCO) of 3000 kDa and incubated at 80 °C for 15 min. The protein was reduced using 5 mM dithiothreitol (DTT) at 60 °C for 30 min and then alkylated in the dark using 10 mM iodoacetamide at room temperature for 45 min. Proteolytic digestion was performed using Trypsin Gold (Promega, USA) at a ratio of 100 parts of protein to 0.5 part of trypsin, followed by incubation at 37 °C overnight. Tryptic digestion and RapiGest activity were terminated by the addition of 1 μL concentrated trifluoroacetic acid (TFA) and incubation of the samples at 37 °C for 20 min. The tryptic peptide solution from each sample was centrifuged at 14000 rpm for 20 min and the resulting supernatants were collected and kept at −80 °C until subsequent analysis^62^.

### LC-MS/MS analysis

The nano LC-MS/MS analysis was performed using an Orbitrap Fusion Tribrid mass spectrometer (Thermo Scientific, USA) as detailed in Yang *et al*.^62^. The samples (2 μL containing 2 μg peptides) were injected and separated on an EASY-nLC 1000 (Dionex, Thermo Scientific, USA) equipped with an Easy-Spray Column Acclaim PepMap™ C18 100 Å (2 μm, 50 μm × 15 cm, Thermo Scientific, USA). Samples were separated by a gradient of 5% to 40% acetonitrile (ACN) with 0.1% formic acid (FA) for 91 min followed by a wash gradient of 85% ACN with 0.1% FA for 6 min and then equilibrated back to 5% ACN with 0.1% FA in 1 min and maintained until subsequent sample injection^62^. The flow rate was set at 250 nL/min. The mass spectrometer was operated in a positive ion mode with a nanospray voltage of 1.5 kV and a source temperature of 250 °C. The instrument was operated in a data dependent acquisition (DDA) mode with a survey MS scan by Orbitrap MS (OTMS) using the following parameters: mass range of 310–1800 m/z with resolving power of 120000, automatic gain control (AGC) of 400000 and a maximum injection time of 50 ms. Top Speed Mode of 3 seconds was used in the selection of precursors with monoisotopic charge state of 2 to 7. These precursors were further analysed in the MS/MS scan. All precursors were filtered using a 20-second dynamic exclusion window and intensity threshold of 5000. The MS/MS spectra was analysed via ion trap MS (ITMS) with the following parameter: rapid scan rate with resolving power of 60000, AGC of 100, isolation window of 1.6 m/z and maximum injection time of 250 ms. Precursors were then fragmented by collision-induced dissociation (CID) and high-energy collision dissociation (HCD) at normalized collision energy of 30% to 28%.

### Protein identification and screening of differently expressed proteins

Raw data were processed using Thermo Scientific™ Proteome Discoverer™ Software v2.1 with the SEQUEST® HT search engine. The MS ion intensities were calculated based on the accurate mass and time tag strategy^62^. The accurate alignment of detected LC retention time and m/z value across different analyses and the area under chromatographic elution profiles of the identified peptides can be compared between different samples. For protein identification, the peptide identification data was compared with the Uniprot® *K*. *pneumoniae* database with a 1% strict FDR and 5% relax FDR criteria using Percolator®. Search parameters were set up to two mis-cleavage with fixed amino acid modification through carbamidomethylation and variable modification through methionine oxidation, together with asparagine and glutamine deamidation. A fragment tolerance of 0.6 Da and a precursor tolerance of 10 ppm were used with trypsin as a digestion enzyme. Identified proteins with at least two unique peptides implied a greater confidence of protein identity. Protein quantification and statistical analyses were performed using Perseus Software v1.6.0.7 (Max Planck Institute of Biochemistry). Each control and treated sample consisted of three biological replicates with three technical replicates, each analysed by LCMS/MS. The protein file with three technical replicates in txt. format from Proteome Discoverer™ were uploaded to the Perseus system for further comparative analysis between samples. The data were log2-transformed to stabilise the variance and scale-normalised to the same mean intensity across the technical replicates. The mean value for all three technical replicates of the same biological samples were grouped together in the same matrix and valid values were obtained by filtering with ‘at least two’, eliminating proteins which only existed in one of the technical replicates. Finally all biological replicates of the same treatment group were consolidated under the same matrix, with the missing values imputed with the random numbers that are drawn from a normal distribution. The histograms were plotted to get a profile for similarity comparison of the ratio for all the samples. Differentially expressed protein between control and treatment were detected using a T-test, the *p*-value were also adjusted for multiple-testing using the permutation-based false discovery rate, with a number of randomization of 250. Proteins were considered to be significantly differentially expressed between treatment groups with an adjusted *q*-value < 0.05 and a fold change ≤−1 or ≥1.

### LVO treatment, RNA extraction and cDNA synthesis

KPC-KP cell cultures were treated with LVO or vehicle buffer prior to RNA extraction. The LVO final concentration used was determined in the checkerboard assay. Both treatment groups had a final volume of 50 mL, and contained Tween 80 at final concentration of 10% to enhance the solubility of LVO. The standard KPC-KP cell inoculum was 1 × 10^5^ cfu/mL. Samples were incubated at 37 °C with shaking at 200 rpm followed by RNA extraction using the TransZol RNA purification kit (Transgen Biotech, China). Treatment time was as determined in the time kill analysis. RNA (0.5 ng) was subjected to reverse transcription with QuantiNova Reverse Transcription Kit (QIAGEN, Germany) in a 20-μl reaction volume. The synthesized complementary DNA (cDNA) was stored at −20 °C until further use.

### Proteomic expression validation through qRT-PCR analysis

The RNA abundance for several upregulated proteins from LVO-treated KPC-KP was determined by qRT-PCR using QuantiNova SYBR Green PCR (QIAGEN, Germany) on CFX96 Touch Real-Time PCR Detection System (Bio-Rad Laboratories, Inc, USA) as detailed in Yang *et al*.^62^. The Livak method was employed to assess the relative expression of four upregulated genes, namely *deoB*, *fabA*, *udp* and *zapB* and two housekeeping genes, namely *16S rRNA* and *OmpK36* porin. The thermal cycling conditions were as follows: 95 °C for 2 min, followed by 40 cycles of 95 °C for 5 s, and 60 °C for 10 s. In all experiments, no template reactions were used as negative controls. Reactions were performed in triplicate and data were analysed by using the CFX Manage Software (Bio-Rad).

## Bacterial Membrane Disruption Assays

### Zeta potential measurement

The zeta potential of non-treated and treated KPC-KP cells (with LVO or meropenem alone, and in combination) was quantified using Zetasizer Nano ZS instrument (Malvern Instruments, Malvern, UK)^15^. Concentration of LVO and meropenem used was determined in checkerboard assay while treatment time were determined in the time kill analysis^15^. Treated cells were washed with 0.85% saline at least five times before zeta potential measurement^15^. The experiment was performed in three independent replicate.

### Outer membrane (OM) permeability assay

Outer membrane permeability assay was performed as detailed in Yang *et al*.^15^. OD_600nm_ 0.3 KPC-KP cells were treated with LVO and meropenem alone, and in combination with concentrations and treatment times determined from the checkerboard assay and time kill analysis. Upon completion, the samples were washed with 0.85% saline (five times) in order to remove the treatment, and then divided into two equal portions of 10 mL^15^. SDS solution at a final concentration of 0.1% was added to one of the portions while 0.85% saline was added to the other^15^. SDS acts as a permeabilizing probe that causes cell death when sudden influx occurred that can be measured in terms of OD_600nm_ at intervals of 0, 5, 10, 30 and 60 min using a spectrophotometer^15,21^. The assay was completed in three independent replicate.

### Ethidium bromide influx/efflux assay

The assay was performed as described by Viveiros *et al*. (2010) with slight modification^63^. Overnight cultures of KPC-KP was washed and adjusted to OD_600nm_ of 0.5 with MHB. Cells were then distributed to three groups; one group consists of non-treated KPC-KP cells and two groups are LVO-treated KPC-KP cells. Non-treated KPC-KP cells were supplemented with ethidium bromide at final concentration of 1 µg/mL whereas LVO-treated KPC-KP cells were supplemented with LVO and ethidium bromide at a final concentration of 0.63% and 1 µg/mL respectively. The accumulation of ethidium bromide was measured using EnSight multimode plate reader (Perkin Elmer, Massachusetts, USA) at excitation wavelength of 530 nm and emission wavelength of 585 nm every 5 min up to 1 h at 37 °C. Subsequently, cells were harvested and washed with MHB at least 2 times. Fresh MHB supplemented with 0.5% glucose without ethidium bromide was added to the non-treated KPC-KP cells. One of the LVO-treated KPC-KP groups were reconstituted with MHB supplemented with 0.5% glucose and 0.63% LVO while the second group of the LVO-treated KPC-KP cells were reconstituted with MHB supplemented with 0.5% glucose only. Ethidium bromide efflux was again measured at the wavelength mentioned above for another 1 h every 5 min at 37 °C. The assay was completed in triplicate.

### Scanning electron microscopy

KPC-KP cells were treated with LVO and meropenem alone, and in combination with concentrations and treatment times determined from the checkerboard assay and time kill analysis. Harvested KPC-KP cells were washed with 0.85% (w/v) saline five times. Sample preparation for scanning electron microscopy was performed as detailed in Yang *et al*.^15^. Samples were fixed with 4% glutaraldehyde for 5 h and 1% osmium tetroxide for 2 h at 4 °C^15^. The samples were fully dehydrated with increasing concentrations of acetone (35–95%) for 10 min followed by 100% acetone for 15 min for three times^15^. Samples were subjected to critical point drying for 30 min (BalTec CPD 030, Bal-Tec, Balzers, Liechtenstein) and secured onto the specimen stub using double sided tape. Lastly, samples were sputter-coated with gold using a cool sputter coater (BalTec SCD 005) and observed via a JEOL JSM-6400 instrument (JEOL, Tokyo, Japan) at 15 kV^15^.

### ROS measurement

ROS measurement was performed as detailed in^38^. KPC-KP cell treatment was as detailed in time kill assay involving non-treated and LVO-treated group only. KPC-KP cell was pelleted using centrifugation at 10000 rpm for 5 min and washed with PBS. Non-treated, LVO-treated and H_2_O_2_-treated KPC-KP cells were treated with 20 μM of DCF-DA for 30 min at 37 °C. After incubation, the cell pellet was collected via centrifugation at 10000 rpm for 5 min with supernatant removed and resuspended in PBS. Fluorescence intensity was quantified immediately at an excitation and emission wavelengths of 485 and 528 nm using Tecan microplate reader (Tecan Trading AG, Switzerland). Fluorescent reading of all three groups were normalized with their respective constituent in the absence of KPC-KP cells. The assay was completed in triplicate.

### Lipid peroxidation assay

Lipid peroxidation assay was performed as detailed in^38^. KPC-KP cell treatment was as detailed in time kill assay involving non-treated and LVO-treated group only. In addition, a positive control of KPC-KP cells treated with H_2_O_2_ were also included. Cell pellet was collected using centrifugation at 10000 rpm for 5 min and washed with phosphate-buffered saline (PBS). Supernatant collected was termed treatment media. Resulting cell pellet was then sonicated as detailed in protein extraction section with supernatant collected and termed cell lysate. Both treatment media and cell lysate were subjected to MDA measurement by mixing 500 µL of either treatment media and cell lysate to 400 µL of 15% trichloroacetic acid and 800 µL of 0.67% TBA in 0.01% butylated hydroxytoluene. Samples were then vortex and incubated at 95 °C for 20 minutes in a water bath. A volume of 3 mL of butanol was added into the sample followed by gentle mixing. A volume of 200 µL of the butanol phase was collected from each sample with absorbance measured at 532 nm. The amount of MDA present was estimated using MDA standard curve and normalized based on protein concentration of each sample. The assay was completed in triplicate.

### GC-MS

The GC-MS analysis was performed as detailed in Yang *et al*. (2018), using an Agilent GC-MS, 7890A GC System with a triple-axis detector (5975C MSD) and an HP-5MS column (30 m × 250 μm × 0.25 μm) (Agilent Technologies, California, USA)^33^. Helium was the carrier gas in the MS. LVO was injected with an auto-injector heated to 250 °C (Agilent Technologies 7693 Auto-sampler, California, USA). The oven column was temperature-programmed from 40 °C (2 min) to 175 °C at a rate of 5 °C min^−1^ within 10 min^33^. The temperature was increased to 250 °C at a rate of 90 °C min^−1^ within the next 5 min^33^. The flow rate of the column was 1 mL min^−1^ with a split ratio of 40:1. EI mode with scan range 30–450 m/z was used to analyze the MS spectrum. The temperature of the MS source was 250 °C whereas the MS quad was 150 °C. Identification of compounds was solely based on the comparison of the mass spectra with those in National Institute of Standards and Technology libraries. The relative percentage of the identified compounds was computed from their GC peak area and only components with total abundance exceeding 0.1% were presented^33^.

## Supplementary information

Dataset 1

## Data Availability

All data generated or analysed during this study are included in the Supplementary Information files.
