# Supplementary material for: Lavender essential oil induces oxidative stress which modifies the bacterial membrane permeability of carbapenemase producing Klebsiella pneumoniae
Source: Sci Rep. 2020 Jan 21;10:819. doi: 10.1038/s41598-019-55601-0 (PMC6972767; doi:10.1038/s41598-019-55601-0)
Supplement: Supplementary file 1 — Dataset 1 [file 41598_2019_55601_MOESM1_ESM.zip › Supplementary Information/Supplementary Figure S1-3 and Tables S1-2.docx]

**Lavender essential oil induces oxidative stress which modifies the bacterial membrane permeability of carbapenemase producing *Klebsiella pneumoniae***

**Shun-Kai Yang**^1^, Warren Thomas^2^, Riaz Akseer^3^, Maryam Sultan Alhosani^3^, Aisha Abushelaibi^3^, Swee-Hua-Erin Lim^2,3^*, Kok-Song Lai^1^*.

^1^Department of Cell and Molecular Biology, Faculty of Biotechnology and Biomolecular Sciences, Universiti Putra Malaysia, 43400 Serdang, Selangor, Malaysia.

^2^Perdana University-Royal College of Surgeons in Ireland School of Medicine, Perdana University, MAEPS Building, 43400, Serdang, Selangor, Malaysia.

^3^Health Sciences Division, Abu Dhabi Women’s College, Higher Colleges of Technology, 41012 Abu Dhabi, United Arab Emirates.

.

*** Correspondence:**

Kok-Song Lai

[laikoksong@upm.edu.my](mailto:laikoksong@upm.edu.my)

Swee-Hua-Erin Lim

[lerin@hct.ac.ae](mailto:lerin@hct.ac.ae)


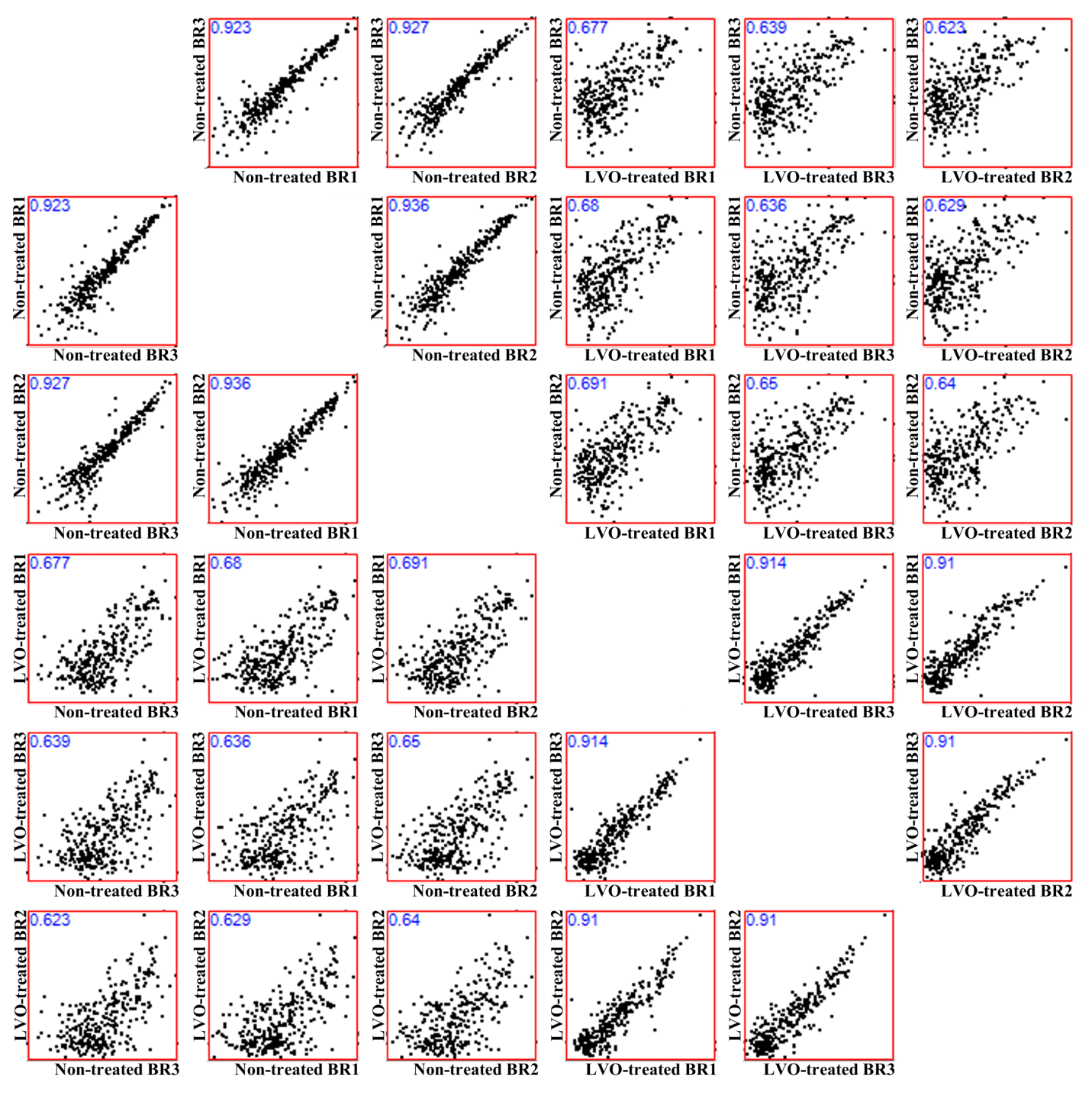


**Supplementary Figure S1.** Scatter plot with Pearson correlation value between biological and independent replicates of non-treated and LVO-treated proteome profile. BR refers to biological replicate.

**
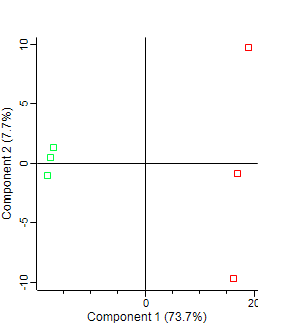
**

**Supplementary Figure S2.** Principle component analysis of non-treated (designated with green box) and LVO-treated (designated with red box) treated KPC-KP cells.


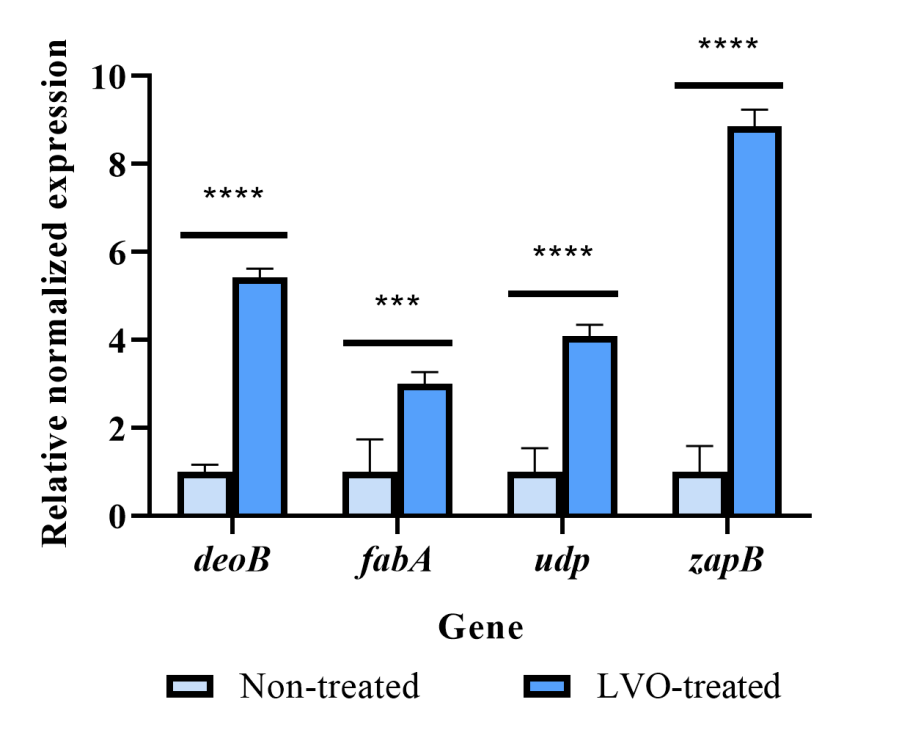


**Supplementary Figure S3.** Expression patterns of *deoB, fabA, udp and zapB* genes in KPC-KP cells subjected to LVO treatment. Results are presented as differential relative transcript abundance. Data were analyzed by one-way ANOVA; ***=p<0.001, ****=p<0.0001.

| Gene | Sequence | Efficiency (%) |
| --- | --- | --- |
| F-*16S rRNA* | CGGCCGGGAACTCAAAGGAG | 91.0 |
| R-*16S rRNA* | AGAGAAGCGACCTCGCGAGA |  |
| F-*deoB* | CTGGTGAAAGCGCATGAAGG | 93.5 |
| R-*deoB* | AGGCCAAAGGTCTCTTCGTG |  |
| F-*fabA* | CTACCGCATCCACTTCAA | 90.3 |
| R-*fabA* | ATCTGAAAGTGGGTCTGTTC |  |
| F-*udp* | CCTCGGATACCTTCTACC | 93.4 |
| R-*udp* | TATGAAATGGAATCCGCC |  |
| F-*zapB* | TAAGGTTCAGCAGGCGATTGA | 97.3 |
| R-*zapB* | AGACTTCTTCCATGCGACCC |  |
| F-*OmpK36* | CGGTTACGGCCAGTGGGAATA | 103.9 |
| R-*OmpK36* | GGACCGACGTTCTGCCGGAATT |  |

**Supplementary Table S1.** List of primers used in this study. F refers to forward primer while R refers to reverse primer.

| No | Library/ID | Retention time | Composition (%) | CAS no. | Retention index | Chemical characterization | Antioxidant/prooxidant activity |
| --- | --- | --- | --- | --- | --- | --- | --- |
| 1 | Linalyl anthranilate | 18.10 | 45.9 | 7149-26-0 | 1258.99 | Terpene | NA |
| 2 | Linalool | 13.60 | 34.5 | 78-70-6 | 1102.78 | Terpene alcohol | Antioxidant and prooxidant |
| 3 | Β-caryophyllene | 26.40 | 2.4 | 1139-30-6 | 1589.08 | Sesquiterpene | Antioxidant |
| 4 | Borneol | 15.50 | 1.9 | 507-70-0 | 1167.79 | Terpene derivative | Antioxidant |
| 5 | Longicyclene | 22.40 | 1.7 | 1137-12-8 | 1423.42 | Sesquiterpene | NA |
| 6 | Hexyl isobutyrate | 16.20 | 1.6 | 2349-07-7 | 1193.04 | Ester of isobutyric acid | NA |
| 7 | Ethyl 2-(5-methyl-5-vinyltetrahydrofuran-2-yl) propan-2-yl carbonate | 12.70 | 1.2 | 1000373-80-3 | 1073.28 | Carbonate ester | NA |
| 8 | Camphor | 14.80 | 0.7 | 76-22-2 | 1145.33 | Terpenoid | Antioxidant |
| 9 | 3-octanone | 10.10 | 0.7 | 106-68-3 | 987.59 | Ketone | NA |
| 10 | 3-hexenyl butyrate | 20.30 | 0.7 | 16491-36-4 | 1341.68 | Ester of butyric acid | NA |
| 11 | 1-Octen-1-ol, acetate | 13.90 | 0.6 | 77149-68-9 | 1113.52 | Ester of acetic acid | NA |
| 12 | Cyclohexanepropanol, 2-acetoxy- | 20.70 | 0.5 | 1000197-25-8 | 1356.76 | Alcohol derivative | NA |
| 13 | Limonene oxide | 20.60 | 0.5 | 4959-35-7 | 1353.88 | Monoterpenoid | Antioxidant |
| 14 | (R)-lavandulyl acetate | 19.00 | 0.4 | 1000360-39-6 | 1292.05 | Acetate ester | - |
| 15 | Acetic acid | 10.90 | 0.4 | 142-92-7 | 1015.26 | Acid | Antioxidant and prooxidant |
| 16 | Geranyl acetate | 21.40 | 0.3 | 105-87-3 | 1385.36 | Monoterpene | Prooxidant |
| 17 | Β-farnesene | 23.30 | 0.3 | 77129-48-7 | 1458.78 | Sesquiterpene | NA |
| 18 | Camphene | 8.80 | 0.3 | 79-92-5 | 946.91 | Monoterpene | Antioxidant |
| 19 | 5-Ethyl-3-methylhept-1-en-4-ol | 10.40 | 0.2 | 286424-80-4 | 997.51 | Alcohol derivative | NA |
| 20 | Trans-linalool oxide | 15.70 | 0.2 | 39028-58-5 | 1176.34 | Furanoid | NA |
| 21 | 1-Octen-3-ol | 9.90 | 0.2 | 3391-86-4 | 981.27 | Alchohol | NA |
| 22 | Neryl acetate | 20.90 | 0.2 | 141-12-8 | 1366.30 | Acetate ester | NA |
| 23 | Hexanoic acid | 21.50 | 0.2 | 6378-65-0 | 1386.65 | Acid | NA |
| 24 | Pyrrolidines | 24.90 | 0.2 | 1000301-18-1 | 1523.32 | Amine | Antioxidant and prooxidant |
| 25 | Trans-α-bergamotene | 22.80 | 0.2 | 13474-59-4 | 1438.64 | Sesquiterpene | NA |
| 26 | p-Cymene | 11.20 | 0.2 | 99-87-6 | 1024.62 | Monoterpene | Antioxidant |
| 27 | (-)-bornyl acetate | 17.20 | 0.2 | 5655-61-8 | 1229.70 | Acetate ester | Antioxidant |
| 28 | m-cymen-8-ol | 16.00 | 0.2 | 5208-37-7 | 1184.89 | Monoterpene alcohol | NA |
| 29 | Hotrienol | 24.30 | 0.1 | 29957-43-5 | 1500.74 | Alcohol derivative | NA |
| 30 | P-cymen-8-ol | 16.10 | 0.1 | 1197-01-9 | 1188.07 | Monoterpene alcohol | NA |
| 31 | Propanoic acid | 15.00 | 0.1 | 2349-07-7 | 1150.10 | Acid | NA |

**Supplementary Table S2.** Chemical composition of LVO via GC-MS analysis. NA: No studies regarding antioxidant and prooxidant was reported.
