# Supplementary material for: Lavender essential oil induces oxidative stress which modifies the bacterial membrane permeability of carbapenemase producing Klebsiella pneumoniae
Source: Sci Rep. 2020 Jan 21;10:819. doi: 10.1038/s41598-019-55601-0 (PMC6972767; doi:10.1038/s41598-019-55601-0)
Supplement: Supplementary file 1 — Dataset 1 [file 41598_2019_55601_MOESM1_ESM.zip › Supplementary Information/Supplementary Figure S1-3 and Tables S1-2.pdf]

Lavender essential oil induces oxidative stress which modifies the bacterial membrane permeability of carbapenemase producing *Klebsiella pneumoniae*

Shun-Kai Yang[1], Warren Thomas[2], Riaz Akseer[3], Maryam Sultan Alhosani[3], Aisha Abushelaibi[3], Swee-Hua-Erin Lim[2,3]*, Kok-Song Lai[1]*.

[1]Department of Cell and Molecular Biology, Faculty of Biotechnology and Biomolecular Sciences, Universiti Putra Malaysia, 43400 Serdang, Selangor, Malaysia.

[2]Perdana University-Royal College of Surgeons in Ireland School of Medicine, Perdana University, MAEPS Building, 43400, Serdang, Selangor, Malaysia.

[3]Health Sciences Division, Abu Dhabi Women's College, Higher Colleges of Technology, 41012 Abu Dhabi, United Arab Emirates.

.

*** Correspondence:**

Kok-Song Lai

laikoksong@upm.edu.my

Swee-Hua-Erin Lim

lerin@hct.ac.ae

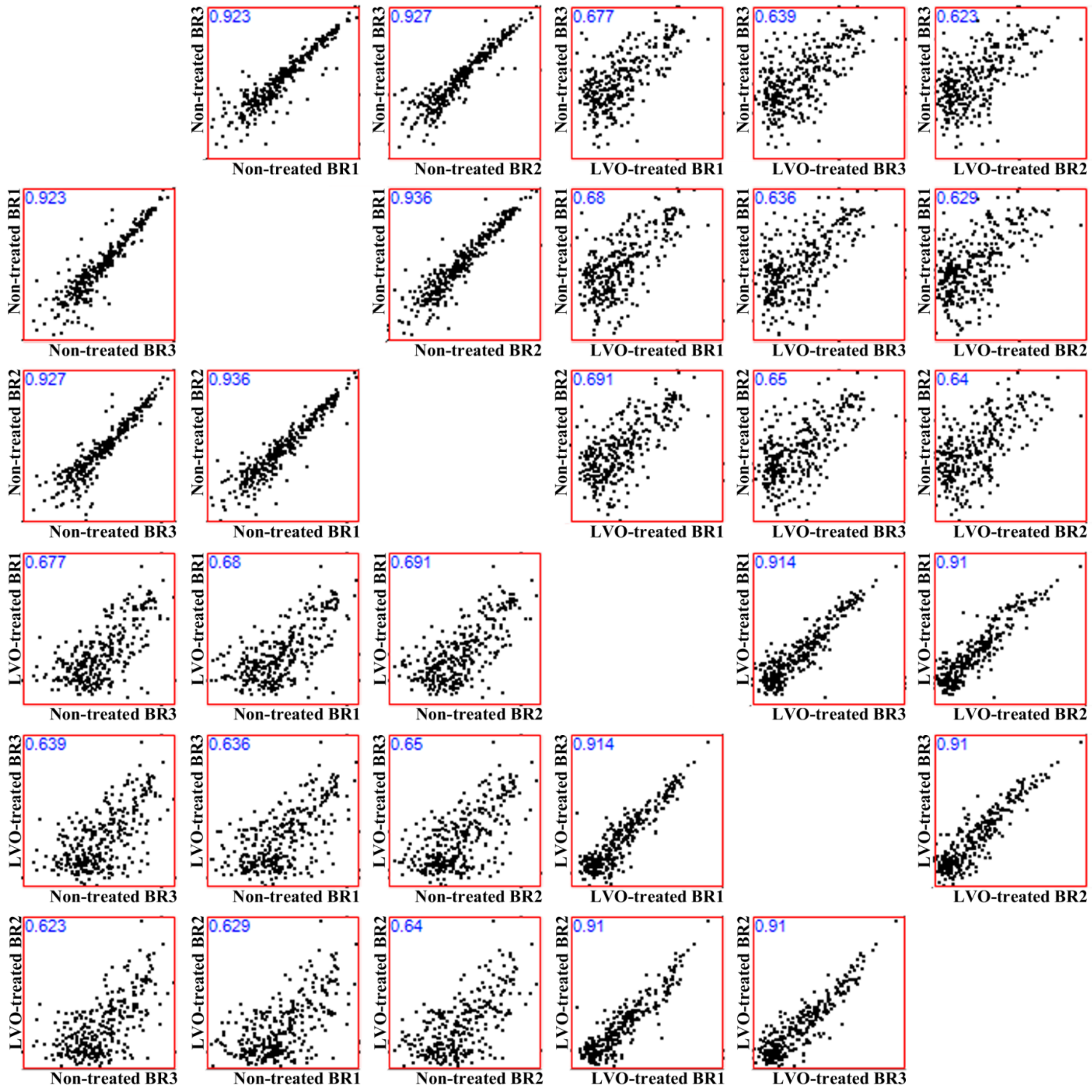

Supplementary Figure S1. Scatter plot with Pearson correlation value between biological and independent replicates of non-treated and LVO-treated proteome profile. BR refers to biological replicate.

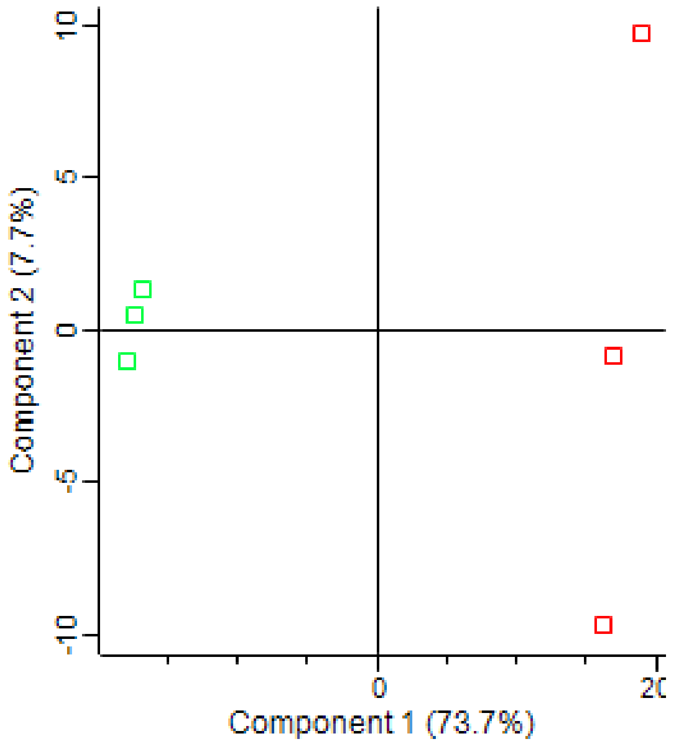

Supplementary Figure S2. Principle component analysis of non-treated (designated with green box) and LVO-treated (designated with red box) treated KPC-KP cells.

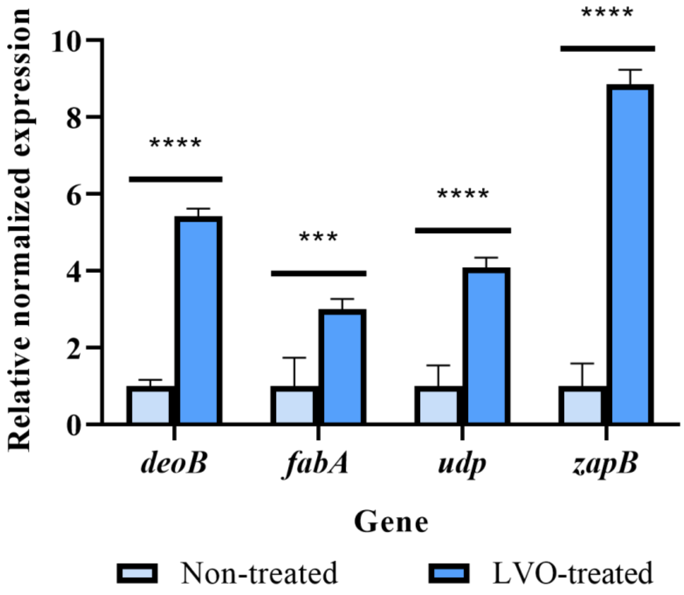

Supplementary Figure S3. Expression patterns of *deoB, fabA, udp and zapB* genes in KPC-KP cells subjected to LVO treatment. Results are presented as differential relative transcript abundance. Data were analyzed by one-way ANOVA; ***=p<0.001, ****=p<0.0001.

Gene	Sequence	Efficiency (%)
F-*16S rRNA*	CGGCCGGGAACTCAAAGGAG	91.0
R-*16S rRNA*	AGAGAAGCGACCTCGCGAGA	
F-*deoB*	CTGGTGAAAGCGCATGAAGG	93.5
R-*deoB*	AGGCCAAAGGTCTCTTCGTG	
F-*fabA*	CTACCGCATCCACTTCAA	90.3
R-*fabA*	ATCTGAAAGTGGGTCTGTTC	
F-*udp*	CCTCGGATACCTTCTACC	93.4
R-*udp*	TATGAAATGGAATCCGCC	
F-*zapB*	TAAGGTTCAGCAGGCGATTGA	97.3
R-*zapB*	AGACTTCTTCCATGCGACCC	
F-*OmpK36*	CGGTTACGGCCAGTGGGAATA	103.9
R-*OmpK36*	GGACCGACGTTCTGCCGGAATT	

Supplementary Table S1. List of primers used in this study. F refers to forward primer while R refers to reverse primer.

No	Library/ID	Retention time	Composition (%)	CAS no.	Retention index	Chemical characterization	Antioxidant/prooxidant activity
1	Linalyl anthranilate	18.10	45.9	7149-26-0	1258.99	Terpene	NA
2	Linalool	13.60	34.5	78-70-6	1102.78	Terpene alcohol	Antioxidant and prooxidant
3	B-caryophyllene	26.40	2.4	1139-30-6	1589.08	Sesquiterpene	Antioxidant
4	Borneol	15.50	1.9	507-70-0	1167.79	Terpene derivative	Antioxidant
5	Longicyclene	22.40	1.7	1137-12-8	1423.42	Sesquiterpene	NA
6	Hexyl isobutyrate	16.20	1.6	2349-07-7	1193.04	Ester of isobutyric acid	NA
7	Ethyl 2-(5-methyl-5-vinyltetrahydrofuran-2-yl) propan-2-yl carbonate	12.70	1.2	1000373-80-3	1073.28	Carbonate ester	NA
8	Camphor	14.80	0.7	76-22-2	1145.33	Terpenoid	Antioxidant
9	3-octanone	10.10	0.7	106-68-3	987.59	Ketone	NA
10	3-hexenyl butyrate	20.30	0.7	16491-36-4	1341.68	Ester of butyric acid	NA
11	1-Octen-1-ol, acetate	13.90	0.6	77149-68-9	1113.52	Ester of acetic acid	NA
12	Cyclohexanepropanol, 2-acetoxy-	20.70	0.5	1000197-25-8	1356.76	Alcohol derivative	NA
13	Limonene oxide	20.60	0.5	4959-35-7	1353.88	Monoterpenoid	Antioxidant
14	(R)-lavandulyl acetate	19.00	0.4	1000360-39-6	1292.05	Acetate ester	-
15	Acetic acid	10.90	0.4	142-92-7	1015.26	Acid	Antioxidant and prooxidant
16	Geranyl acetate	21.40	0.3	105-87-3	1385.36	Monoterpene	Prooxidant
17	B-farnesene	23.30	0.3	77129-48-7	1458.78	Sesquiterpene	NA
18	Camphene	8.80	0.3	79-92-5	946.91	Monoterpene	Antioxidant
19	5-Ethyl-3-methylhept-1-en-4-ol	10.40	0.2	286424-80-4	997.51	Alcohol derivative	NA

20	Trans-linalool oxide	15.70	0.2	39028-58-5	1176.34	Furanoid	NA
21	1-Octen-3-ol	9.90	0.2	3391-86-4	981.27	Alchohol	NA
22	Neryl acetate	20.90	0.2	141-12-8	1366.30	Acetate ester	NA
23	Hexanoic acid	21.50	0.2	6378-65-0	1386.65	Acid	NA
24	Pyrrolidines	24.90	0.2	1000301-18-1	1523.32	Amine	Antioxidant and prooxidant
25	Trans-α-bergamotene	22.80	0.2	13474-59-4	1438.64	Sesquiterpene	NA
26	p-Cymene	11.20	0.2	99-87-6	1024.62	Monoterpene	Antioxidant
27	(-)-bornyl acetate	17.20	0.2	5655-61-8	1229.70	Acetate ester	Antioxidant
28	m-cymen-8-ol	16.00	0.2	5208-37-7	1184.89	Monoterpene alcohol	NA
29	Hotrienol	24.30	0.1	29957-43-5	1500.74	Alcohol derivative	NA
30	P-cymen-8-ol	16.10	0.1	1197-01-9	1188.07	Monoterpene alcohol	NA
31	Propanoic acid	15.00	0.1	2349-07-7	1150.10	Acid	NA

Supplementary Table S2. Chemical composition of LVO via GC-MS analysis. NA: No studies regarding antioxidant and prooxidant was reported.
